# The Correlation Between Progesterone and Mammographic Density in Postmenopausal Women: A Systematic Review of the Literature and Meta-Analysis

**DOI:** 10.7759/cureus.45597

**Published:** 2023-09-20

**Authors:** Ioannis Boutas, Adamantia Kontogeorgi, Nektarios I Koufopoulos, Abraham Pouliakis, Constantine Dimitrakakis, Dionysios T Dimas, Kyparissia Sitara, Sophia Kalantaridou, Fatih Durmusoglu

**Affiliations:** 1 Breast Unit, Rea Maternity Hospital, Athens, GRC; 2 Third Department of Obstetrics and Gynecology, National and Kapodistrian University of Athens, Athens, GRC; 3 Second Department of Pathology, National and Kapodistrian University of Athens, Athens, GRC; 4 Breast Unit, First Department of Obstetrics and Gynecology, National and Kapodistrian University of Athens, Athens, GRC; 5 Breast Unit, Athens Medical Center, Psychiko Clinic, Athens, GRC; 6 Department of Internal Medicine, “Elpis” General Hospital of Athens, Athens, GRC; 7 Department of Obstetrics and Gynecology, Istanbul Medipol International School of Medicine, Istanbul, TUR

**Keywords:** estrogen, postmenopausal, mammographic density, breast cancer, progesterone

## Abstract

Higher mammographic breast density in premenopausal and postmenopausal women is related to a higher breast cancer risk. In this review, we analyze the correlation between estrogen, progesterone, and mammographic density in postmenopausal women and clarify whether these findings are consistent across different types of mammographic breast density. We extracted data concerning mammographic density increases in the populations treated with estrogen-only hormone replacement therapy and those treated with estrogen and progestin hormone replacement therapy. Postmenopausal women treated with estrogen and progesterone regimens had a statistically significant lesser mammographic density increase than estrogen-only hormone replacement therapy regimens.

## Introduction and background

Higher mammographic breast density in premenopausal and postmenopausal women signifies a higher breast cancer risk and development [1-3]. When examining breasts in a mammogram, specialists view the amount of connective, epithelial, and fat tissue in the breast [4]. The radiolucent (darker) areas of the mammogram primarily consist of fat, while the non-radiolucent (lighter) areas indicate the glandular and fibrous tissues [5-8].

Women with a breast density area of ≥75% have a three-to-six-fold risk of developing breast cancer compared to women with a breast density area of ≤5% [5,9]. Premenopausal women have higher-density breasts since mammographic breast density decreases with age [10]. The measurement of absolute mammographic density is the measurement of dense areas composed of epithelial and stromal tissues on the breast. Absolute and percent mammographic densities positively correlate with the risk of malignant transformation. Higher mammographic density increases the risk of developing breast cancer [1-3]. Interestingly, various breast cancer risk factors, such as BMI and age, have been associated with different density compositions [6-8].

In postmenopausal women, progesterone promotes epithelial and stromal growth in the mammary gland and is associated positively with the increased risk of developing breast cancer [11]. Various studies associated reduced mammographic density percentage with administered tamoxifen [12] and increased mammographic density percentage with administered progesterone [13,14]. However, it is unclear whether progesterone levels throughout the menstrual cycle are associated with mammographic density or vary by mammographic density phenotypes [8,15,16].

The main objective of this review is to analyze the correlation between estrogen, progesterone, and mammographic density in postmenopausal women and clarify whether these findings are consistent across different types of mammographic breast density. To achieve this, we found six articles from the relevant bibliography. We extracted the relevant patient characteristics and estrogen/progesterone hormonal replacement therapy (HRT) data correlating to mammographic density. With the help of a review tool, we analyzed the data. We attempted to determine whether there is a correlation between estrogen, progesterone, various hormonal replacement therapies, and mammographic density levels in postmenopausal women.

## Review

Materials and methods

Search Strategy

The review followed the Preferred Reporting Items for Systematic Reviews and Meta-Analyses (PRISMA) guidelines (http://www.prismastatement.org/; accessed on August 12th, 2023).

We searched for ((“progesterone” OR “progestogen”) OR (“estrogen” OR “estrogens”) AND (“mammary” OR “mammography” OR “mammographic” OR “mammary glands” OR “breast” OR “breasts”) AND (“postmenopause” OR “postmenopausal” OR “postmenopausal women”) AND (“mammographic density”)). The search on PubMed yielded (all fields; 172 results; https://pubmed.ncbi.nlm.nih.gov, accessed on August 12th, 2023), Scopus (Title/Abstract/Keywords; 212 results; https://www.scopus.com/, accessed on August 12th, 2023), and Web of Science (all fields, 378 results; https://login.webofknowledge.com, accessed on August 12th, 2023). For our included studies, only those published in the last 20 years were considered relevant and up-to-date. This decision was taken to ensure consistency in evidence across the studies. No other restrictions were applied to the query.

The result set consisted of 762 studies independently assessed by two authors (AK and IB) by reading their abstracts. For all relevant articles, the authors reviewed the entire paper, and all inclusion and exclusion criteria were applied to narrow down the result set to only the pertinent studies of interest. Any disagreements were resolved following consensus. Furthermore, the snowball procedure was applied in all selected papers for other studies that might have been relevant.

Inclusion and Exclusion Criteria

The eligible studies considered by the authors were case-control, cross-sectional, and cohort studies that examined mammographic density, progesterone, and/or estrogen HRT in postmenopausal women. Two authors (AK and IB) manually assessed the relevant articles to properly report the patients' hormonal replacement therapy, mammographic density reference, and appropriate population characteristics (age, BMI, parental status, smoking, anti-contraceptive consumption, etc.). All cell culture studies, case series, and case reports were considered irrelevant and were excluded from the result set. Any studies that lacked information on density or hormonal therapy treatment were also excluded. Finally, only studies in the English language were considered.

Data Extraction and Quality Assessment

After the inclusion and exclusion criteria were applied to the result set, the result set was narrowed down to six relevant studies. Two authors (AK and IB) independently reviewed the studies and extracted all relevant data of interest using a customized data extraction form. The form included the following characteristics: author, year of publication, number of patients, the mean age of the population group, parity (nulliparous or parous), BMI, smoking habits, mammographic density, and HRT. Based on the extracted characteristics, each study was rated poor, fair, or good, emphasizing the sample size and the appropriate reporting of the outcome variables. The authors also performed a statistical analysis based on the study population and mean breast density using the Review Manager 5.4 Tool (Cochrane Library, Massachusetts, USA, 2022).

Results

Study Characteristics

**Figure 1:**
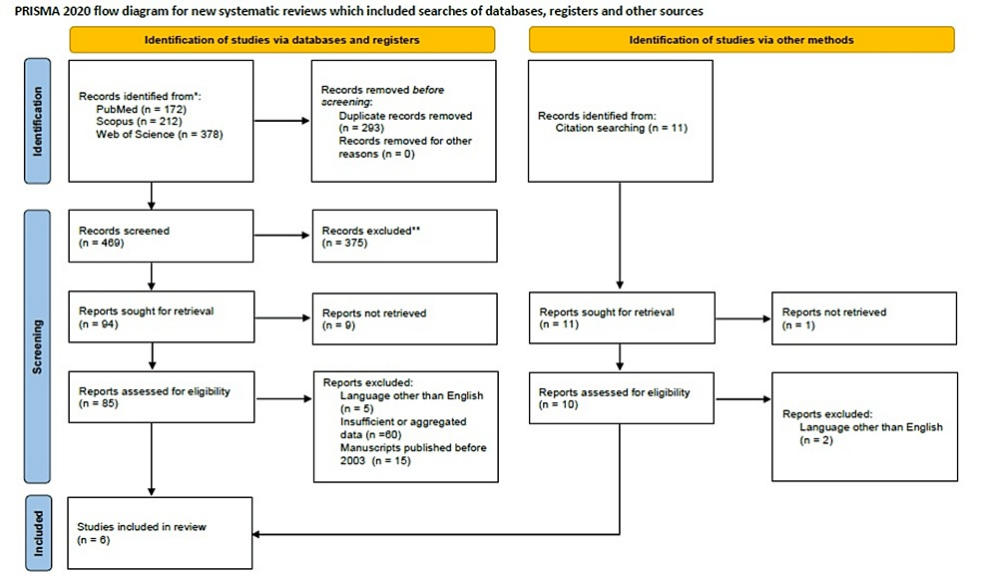
PRISMA diagram of the process followed in order to limit the sample size to the studies of interest. PRISMA: Preferred Reporting Items for Systematic Reviews and Meta-Analyses.

We identified 772 studies eligible for this review, as seen in the PRISMA study design diagram in Figure 1. After removing duplicates (n=293), the two authors that screened the titles and abstracts chose 94 studies as candidates for inclusion. After full-text evaluation, assessment, and application of the criteria predetermined by the authors, 89 more studies were excluded. The snowball procedure applied yielded 11 more relevant papers. Their evaluation excluded 10 of them. Since other reviews are scarce across the bibliography, we included studies from 2003 onwards to present a more suitable and up-to-date conclusion. Therefore, only studies with precise reporting data were considered, including BMI, age, mammographic density, hormonal replacement therapy regimes, and written in English.

The six studies that were identified for inclusion referred to 40649 postmenopausal women [14,17-21]. Most of the studies were conducted in the USA (n=3), with the rest being conducted in Greece (n=1), Spain (n=1), and Turkey (n=1). The study population consisted of various ethnic backgrounds (White, Black/African American, Hispanic/Latino, Asian/Pacific Islander, and others). The general population characteristics of these studies can be seen in Table 1. The mean age and BMI were similar for all studies included in this review. Although we intended to report on anti-contraceptive usage, the studies needed more information to do so.

**Table 1:** General population characteristics. CEE: conjugated equine estrogens; MPA: medroxyprogesterone acetate; MP: micronized progesterone; E2: 17b-estradiol 2 mg; NETA: norethisterone acetate; EPT: estrogen-progestin therapy; ET: estrogen therapy.

Study	Country	Participants (n)	Age (mean)	BMI (mean)	Parity		Smoking?	HRT protocol	Additional comments
Aiello et al. [17]	USA	39296	60.0	27.5	EPT	ET	N/A	20159 (ET)	Postmenopausal women with no history of breast cancer
					6009 (Nulliparous)	2243 (Nulliparous)			
					976 (One child)	774 (One child)		19374 (EPT)	
					31548 (Unknown)	12780 (Unknown)			
Carmona-Sànchez et al. [18]	Spain	165	49.7 ± 6.7	27.3 ± 4.1	28 (Nulliparous)		N/A	38 (ET)	Postmenopausal women treated with HRT for five years
					137 (One or more)			127 (CEE/MPA)	
Christodoulakos et al. [19]	Greece	121	52.2	25.5	N/A		8 (control)	34 (CEE/MPA)	Postmenopausal women who had never received or were past users of hormone replacement therapy
							10 (CEE)	35 (E2/NETA)	
							4 (CEE/MPA)	25 (CEE)	
							9 (E2/NETA)	27 (Not qualified)	
Crandall et al. [20]	USA	428	56.2	25.9	N/A		222 (Never)	323 (EPT)	Postmenopausal women
							51 (Currently)	105 (ET)	
							155 (Former)		
Greendale et al. [14]	USA	571	56.0	26.2	N/A		292 (Never)	113 (CEE)	Postmenopausal women who were enrolled in the postmenopausal estrogen/progestin interventions trial and randomly assigned to receive placebo, daily CEE, daily CEE+MPA cyclic, daily CEE+MPA continuous, or daily CEE+MP
							77 (Currently)	109 (CEE/MPA– cyclical)	
							225 (Former)	121 (CEE/MPA continuous)	
								114 (CE/MP)	
Topal et al. [21]	Turkey	113	50.0 ± 1.66	N/A	2.4 ± 0.9 (CEE/MPA-continuous)		N/A	16 (CEE/MPA-cyclical)	Postmenopausal women on different HRT regimens
					1.7 ± 0.8 (CEE/MPA-cyclical)			60 (CEE/MPA- continuous)	
					2.8 ± 1.2 (ET)			37 (ET)	

We also extracted data presenting mammographic density increases in the populations treated with estrogen-only hormonal replacement therapy and those treated with estrogen and progestin hormone replacement therapy. The data reported on the percentage of increase in mammographic density, and from a first glimpse, we couldn't see estrogen and progestin hormonal replacement regimens presenting many different results in breast density increase compared to the estrogen-only regimens. The extracted mammographic density data can be seen in Table 2.

**Table 2:** Mammographic density in percentage (%) and actual (N) figures in the population by study.

Study	E			E + P		
	N	Mdi (%)	Mdi (N)	N	Mdi (%)	Mdi (N)
Aiello et al. [17]	20159	7.6	1532.08	19374	3.1	600.59
Carmona-Sànchez et al. [18]	38	7.9	3.00	127	25.2	32.00
Christodoulakos et al. [19]	23	2	0.46	54	7.5	4.05
Crandall et al. [20]	105	1.2	1.26	323	4.2	13.56
Greendale et al. [14]	113	1.17	1.32	344	4.14	14.24
Topal et al. [21]	37	2.5	2.50	76	22.5	17.10

We decided to perform a statistical analysis and see if a statistical tool would accurately represent the data and extracted conclusions. We utilized Review Manager 5.4 (RevMan) for the analysis, and we reformed the percentage data to actual population numbers so the representation of the analysis would be as accurate as possible. Because RevMan does not allow decimal numbers, we had to multiply our data by 100 for the software to accurately represent the data and ensure we hadn't rounded any of the extracted data.

**Figure 2:**
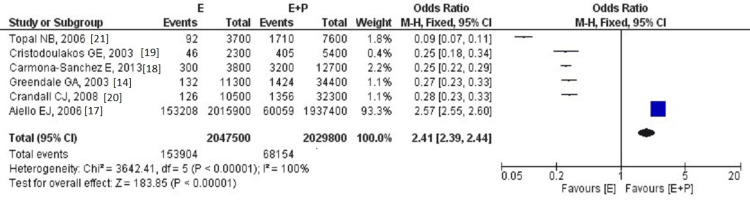
Forest plot indicating mammographic density increase in postmenopausal women following estrogen-only versus estrogen and progestin hormonal replacement therapy. Sources: [14,17-21]. E: estrogen-only group; E+P: estrogen and progestin group.

After introducing the data in RevMan, we observed an obvious, statistically significant result in the forest plot the software produced. Postmenopausal women treated with estrogen and progesterone HRT regimens had lesser mammographic density increases than estrogen-only combined HRT regimens. The forest plot data and results can be seen in Figure 2.

Discussion

In recent years, there has been an emphasis on establishing HRT as a preventive measure to avoid cardiovascular events and osteoporotic fractures [22]. It is crucial for physicians that HRT is a safe option for all female patients. There was intense concern from the scientific community regarding the association of the treatment with thromboembolic events, strokes, and an increased risk for breast cancer [23]. First, the Women's Health Initiative (WHI) study, which was carried out more than 30 years ago, determined there is no increase in breast cancer risk using systemic estrogen-only HRT. However, multiple studies followed, indicating that estrogens have oncogenic action [24]. During that period, different HRT combinations were proposed that could reduce the risk of breast cancer, for example, the addition of androgens, but unfortunately, they did not reach a positive conclusion [25]. One indicator of a woman's response to HRT is changes in mammary gland density. The higher the density, the higher the chance of cancer, hence why many papers examine this correlation [26].

The data from the existing literature demonstrate that HRT, in most cases, can lead to an increase in breast density [27]. More specifically, a comparison has been made in the breast density of women taking HRT and women who have never undergone any during their lifetime, with precise results indicating that the latter group has significantly lower density [17,26]. However, an attempt has yet to be made to perform a meta-analysis between the two populations so that the conclusions drawn can be statistically substantiated.

Azam et al., in 2020, carried out the first extensive literature report on the possible association of HRT with changes in breast density. It summarized the effect of combinations of estrogens and their derivatives with or without the addition of progesterone [28]. This study shows that the combination of estrogen and progesterone is associated with increased breast density. However, the heterogeneity of the populations under consideration and the different parameters considered in each study warrant further research concerning the research question. In the present paper, after an extensive literature review, we considered six studies concerning postmenopausal women who received one year of hormonal therapy either with estrogen alone or in combination with progesterone. From the statistical analysis of the data, a statistically significant conclusion emerges that administering estrogen and progesterone does not increase breast density.

Additionally, as part of the literature review, we studied the addition of dydrogesterone to HRT regimens. Despite the difficulties encountered, there are research papers that conclude the beneficial addition of dydrogesterone to HRT regimens with a prophylactic effect on breast cancer [29]. The proposed mechanism focuses mainly on downregulating the progesterone receptor in the mass gland [30]. In most of the studies, cancer cell lines were given estrogen metabolites or estrogen metabolites combined with dydrogesterone. When progesterone was added, the number of cells decreased [31,32]. Other studies on healthy cells showed that cell proliferation is reduced in the presence of dydrogesterone [32]. Finally, the transdermal route of administration may be more effective than oral administration [32]. In addition, published meta-analyses, including 86,881 patients, show that natural progesterone acts prophylactically against the occurrence of breast cancer, with researchers recommending its inclusion in HRT regimens [30].

## Conclusions

In summary, it is crucial to answer whether the addition of progesterone to estrogenic HRT regimens increases the risk of breast cancer. Our study through the performed meta-analysis shows that the association of progesterone addition is statistically significant as it limits the change in mammary density. Another question is the choice of the appropriate progesterone; although medroxyprogesterone is most preferred in HRT, new literature recognizes dydrogesterone and natural progesterone as possible additions. In any case, primary research and clinical studies are needed to answer the research question. Until then, from the existing literature, progesterone appears to be an ally in preventing the increase in breast density and, possibly, breast cancer. 
